# Notes and Comments

**Published:** 1923-02

**Authors:** 


### NOTES AND COMMENTS.

### THE HOSPITAL SAVING ASSOCIATION.

WE print on another page some account of the progress that is being made in London with the scheme of the Hospital Saving Association. This admirable plan is still in its earliest infancy, but it is already clear that it is capturing the imagination of those to whom the hospitals must in future mainly look for support, and arousing that local patriotism which, so far as the capital is concerned, is normally dormant. Co-operation is at present only tentative—short views are being taken, and the hospitals which have "come in" are not binding themselves beyond the present year; but even the unenthusiastic cannot but be impressed by the promising character of the scheme and the great future which, if thoroughly explained and carefully worked, it clearly possesses. We are as yet in the stage of spade-work and preliminary organisation, and it is of very real importance that the foundations of what we hope will be a splendid and enduring edifice should be well and truly laid.

### THERMS AND EXPLOSIONS.

VOLTAIRE assured us long ago that the Prophet Habakkuk was *capable de tout.* The mantle of *ce coquin* appears to have descended, first, upon the mathematicians who invented the therm to take the place of the old-fashioned, honest cubic foot of gas, and then upon the gas engineer. The therm burned a hole in the pocket of the householder; the gas engineer threatens to burn the unhappy man himself. Whatever the reason—and the culprits are not agreed among themselves about the precise nature of their crime—the domestic gas has of late become more perilous to its consumer, as many recent explosions and semi-poisonings bear witness. Gas-poisoning, as Dr. Waldo, the City Coroner, has just been pointing out to the jury at one of his inquests, has of late become the fashionable form of suicide, owing to the large amount of carbon monoxide now contained in coal gas in general use for lighting, warming, cooking, and as a motor power for engines. If coal gas percolates through a few feet of soil, the characteristic odour, due mostly to the carbon sulphide contained in it, is, as Dr. Waldo reminds us, apt to disappear. Hence the need of giving opportunity and encouragement to the gas companies to examine their street mains, modern heavy traffic being largely responsible for their fracture. Another danger is that of explosion, since one part of coal gas with ten of atmosphere is a dangerous explosive mixture. At the inquest referred to the jury recommended a reduction of the amount of carbon monoxide in the domestic gas. Certainly the matter cannot be left where it is. It concerns both the Board of Trade and the Ministry of Health. The former is a conglomerate institution which includes, we believe, the Archbishop of Canterbury and other exalted personages, who, among them, form a Board which never meets. The only Board which Oliver Twist was able to perceive was a table, and we know how dutifully he bowed to it under the dread compulsion of Mr. Bumble. But if little may be expected from so amorphous an entity, better things may be looked for from the Ministry of Health, which, before it was happily translated, was also a Board. It is the business of that Ministry to see that the British public is not poisoned either by gaseous emanations or botulism, and we look to it with confidence to save us from the modern Habakkuks.

### THE JENNER CENTENARY.

BY an odd coincidence we are marking the centenary of the death of Edward Jenner, if not in the midst, at all events at what we may hope is the tail end, of an outbreak of smallpox. It may well be that Jenner was not the actual "inventor" of vaccination, but it is to him that we owe inoculation as a practical means of preventing what was once a terrible and disfiguring disease. Most great discoveries have been forestalled—Newcomen knew something about steam before Watt, and the doctrine of the "survival of the fittest" occurred to Alfred Russel Wallace at least as early as to Darwin. That the world owes an immeasurable debt of gratitude to Jenner most people will continue to believe, modern controversies notwithstanding. Five decades ago there were 58,614 deaths from smallpox; in the decade ending with 1916 there were only 139. Vast as has been the advance of sanitary science in the interval, so vast a saving of life cannot reasonably be attributed to that alone. It has helped, unquestionably, but all experience shows that the vaccinated, and especially the revaccinated, are safer than those who have not been inoculated. Moreover, the disease is now infinitely less virulent than formerly, and it may not unfairly be surmised that the vaccinated children of the vaccinated may have an increased form of resistance. We are well aware that many opponents of vaccination regard all this as just as heretical as their own views are deemed to be by others, and that medical opinion, as our own columns have recently borne witness, is less unanimous on the subject than formerly; but the balance of opinion is still vastly on the side of Jenner.

### THE DONCASTER SCHEME.

ON another page we print some account of the outstanding features of the Doncaster Regional Planning Scheme, the first project of its kind to be elaborated in this country. The fact that it is a pioneer scheme would alone make it exceedingly interesting, but it is so bold, it covers so large an area, it has been prepared with so much breadth of vision and so prophetic an eye to the future, that it may well be destined to mark a turning-point in our communal history. Doncaster is a pleasant old town, with many fine old houses, and is the natural capital of its district. That district, which is at present chiefly agricultural, has been found to contain coal measures far richer than was formerly suspected, and these measures are gradually being opened up. Unless precautionary steps are taken the inevitable consequence will be that Doncaster will some day become a second Sheffield, blackened and grimed like its prototype. The local authorities who hold sway over the neighbouring 50 villages, covering some 100,000 acres, have therefore taken counsel together and, with the help of experts, have produced what looks like a sound working plan for saving both Doncaster itself and the countryside for several miles around it from the fate which long ago overtook the Black Country. The low-lying land is set aside for industry and, to some extent, for agriculture, and the higher regions for residential purposes and "clean commerce." The various communities would retain their individuality, parks and open spaces would be provided for, historic spots would be preserved and pretty villages saved from vulgarisation. Thus there would be a nice balance between the claims of the industry which is destined to enrich the district and those of health and æsthetics. The public conscience will no longer endure the selfish and indiscriminate creation of slums or the transformation of smiling country into a conglomeration of spoil-banks and slag-heaps. Black business we needs must have, but we are entitled to regulate the method of its expansion and to save the fair face of nature from being wantonly soiled by the hand of man, even if we can do so only by some interference with private rights. The Doncaster report shows, in broad and simple outline, how we may attain an ideal which is certain, when translated into action, not only to save a whole countryside from profanation, but to add materially to the health and happiness of generations who otherwise would some day curse us.

### THE OVERLAPPING OF PUBLIC ASSISTANCE.

MR. GEOFFREY DRAGE, as Chairman of the Denison House Committee on Public Assistance, once more directs attention to the serious overlapping, confusion and waste in our methods of administering "all beneficiary assistance from rates and taxes, whether maintenance or treatment, for which the recipient does not pay or only pays a portion." Where so many authorities are spending public money, and more especially where two or more of them are spending it in much the same direction, overlapping and consequent waste are inevitable. The whole matter requires thoroughly scientific co-ordination. We are spending eight millions a week, partly out of the Exchequer and partly out of the rates, on various forms of assistance —money which has to be found by tax and rate-payers, the majority of whom can ill afford their share of these tremendous imposts. It is only by going systematically to work that we can cure what is rapidly becoming a public scandal. The first necessity is a complete annual return of all expenditure from rates and taxes on direct public assistance, showing clearly the cost of administration. Then as an authority on the subject has declared, with obvious truth, "the first basis of all reform" is a register of beneficiaries. Moreover, there can be no system without a budget, and the Denison House Committee suggests that there should be an annual estimate of expenditure on direct public assistance. Work so extensive as this would seem to require a whole new department to itself—a fearsome prospect indeed were it not that it is only by some such means that the millions at present wasted can be saved. Mr. Drage desires to see a Permanent Commission of Inquiry and Control to concentrate in one authority the functions of the various central departments. But Mr. Drage would go much further than this and would amalgamate existing unions and parishes into a single administrative area. As a preliminary he would transfer the Ministries of Labour and Pensions to the Ministry of Health, which would thus, "for the time being, at any rate," be the one central administrative authority. That would make a decidedly overgrown department, and it would surely be more practical to transfer the financial side or pensions to the Treasury, leaving the Ministry of Health to cover the medical part of the work. Labour matters might surely go to the Home Office.

### CADET CORPS AS HEALTH CENTRES.

SHOULD the War Office carry out its threat to withdraw all financial aid to Cadet Corps, the consequences are likely to be exceedingly unfortunate, while the saving to the country will be only about £40,000 a year. Many cadet companies are raised in poor and "slummy" districts, and it will be very difficult to replace the loss of the Government grant, relatively small though it has been. Nowhere is it more necessary to teach cleanliness, smartness, and discipline than in such places, and a boy's presence in a Cadet Corps is a guaranty that he is learning to be something better than a loafer and a wastrel. He is taught to keep his body clean and his accoutrements in good order, while where these Corps are raised by religious organisations he is taught to keep his mind clean also. The drill and exercises and the time spent annually in camp are valuable helps to health and vigour, yet it is precisely the poor companies which will be hardest hit by the Government's decision. The Public School Corps will be able to maintain themselves without much difficulty, but it is to be feared that many of those that are most needed and most valuable will be destroyed or seriously diminished in numbers, if not in efficiency.

### A RESEARCH CLEARING-HOUSE.

STATISTICS are often a weariness to the flesh, and of the making of them there is no end. But in the right hands they may be picturesque and even alluring, and when they deal with such momentous matters as life and death, health and disease, they are of vital importance. Statistics lose half their value unless they are based upon a wide range of co-ordinated facts, and we therefore feel great sympathy with Dr. R. Dudfield's suggestion to the Royal Statistical Society for the establishment of a clearing-house for research in relation to disease. The number of men who are doing admirable, and not always well requited, research work into the origin, causation, and history of disease, its diagnosis and treatment, is ever growing, and every year the available material increases in volume. Yet the value of much of it is lost because it is neither readily available, nor scientifically co-ordinated. This mass of data needs, as Dr. Dudfield urges, to be brought together from the hospitals, poor-law and other institutions in which it originates, and analysed by a central body of statisticians with medical experience. The latter is an essential proviso. A mere statistician, however accomplished, who was destitute of medical training and experience, would be unlikely to produce results of real value to our knowledge of disease and its prevalence, and of the circumstances surrounding it. Work such as this ought to be a national undertaking. Without it we are labouring if not exactly in the dark, certainly in a very dim light.

### THE UNCEASING ANNUITANT.

MR. KIPLING once wrote a sly little jibe about "One unceasing wife." The Institute of Actuaries has lately been concerning itself about the unceasing annuitant who, sitting at home at ease, chuckling over his quarterly or half-yearly cheque, obstinately refuses to die, and carries confusion into the tables of mortality upon which his annuity was based. The longevity of pensioners is notorious, but they are ever growing older, and the Assurance Companies are beginning to be seriously concerned about this unrestrained vitality. Mr. W. P. Phelps, himself the manager of such a company, in his Presidential Address to the Institute, points out that in the three septennial periods since 1900 the mortality among annuitants has not only been light, but has progressively been growing lighter. The women, of course, are the worst offenders—their mortality is from 10 to 15 per cent. less than that of the experience of the British offices. The men have had more regard for the susceptibilities of the Tables of Mortality. It is true that they also have lived longer, but their tendency to longevity has been less marked, and the improvement has not been so obviously progressive. But there is consolation for the Offices in the fact that if annuitants live longer so do assured persons. The impropriety of demanding more money than the Offices expected to have to pay is balanced by a virtuous continuance in the payment of insurance premiums. Mr. Phelps thinks that the rates will shortly have to be revised all round, since they are clearly out of date. These experiences of the Assurances Offices are a curiously convincing proof of the notable and continuous improvement in the public health which has come about during the last twenty years.

### AGRICULTURE AND HOUSING.

THE town-dweller is apt to think that the decay of English farming is no concern of his. The tiller of the soil, he says to himself, is a born grumbler, and abundance of cheap food can always be obtained from overseas. He takes too short and too narrow a view to see that a dying agriculture must have very serious reactions for him. It means the sending abroad annually of scores of millions of money that are sorely needed at home, with the inevitable ultimate consequence that the cheap food by which he sets so much store will be dear. It is, in fact, dear already, as everybody knows who eats meat and bread. But, looming in the not very distant future, is a far worse consequence than this, since dear food is of comparatively little moment so long as prosperity provides the money with which to buy it. It is from the open countryside that the towns draw their new supplies of sinew and physical energy, and if this storehouse should be destroyed or more seriously injured than it has been already, the health of the towns, which is as much as to say of the nation, will suffer heavily indeed. The population of the rural districts has been dwindling steadily for years past; even the small country towns, those agreeable conjunctions of the rural and the urban, are becoming more thinly peopled. If the State is to remain healthy, in more senses than one, its crowded towns must be balanced by a substantial agricultural population tilling the soil to its most intensive capacity. The real reason for the desertion of the country for the town has been the difficulty of earning a living there in pleasant circumstances. During the last few years the amenities of village life have been greatly increased, but agricultural wages have now fallen seriously and the laying down of arable land to grass is rapidly reducing the volume of labour the farmer needs. When Parliament reassembles the Government will be prepared, no doubt, with an agricultural policy, but it will be of little avail unless the townsman can be brought to understand that the problem of the land is also the problem of his own health and well-being.

### LOWERED GERMAN VITALITY.

PROFESSOR DR. TJADEN, the Principal Medical Officer of Bremen, gives a melancholy account of health conditions in that city during and since the war. The "hunger-blockade," he declares, caused conspicuous emaciation, and there was a heavy increase in the number of deaths from consumption—in 1918 the mortality was almost double that of 1916—and cases of rickets were multiplied. When money and food were once more available, through charitable agencies, after the Armistice, there was a rapid improvement in the health of the city, but the "nervous tension" of the war period has been carried on to the present time. There is now abundance of food, but little money with which to purchase it. Milk is 180, eggs and meat 200, sugar 320, and wheaten bread 120 times dearer than before the war, and the consequence is a "chronic state of starvation." Medical treatment is beyond the means of large numbers of people. "Hospital charges" are 75 times higher than in 1914, and will shortly be higher still; a doctor's consultation fee is £200. When, however, Dr. Tjaden goes on to say that the proportion of venereal diseases is such that, ten years hence, nearly half the population of Bremen will be suffering from them, he appears to be leaving the domains of fact and science for the risky realms of speculation. And when he seeks to show that these disasters are the result of the Treaty of Versailles, he surely forgets that it was the overweening ambition of Germany and its brutal methods of waging war against peoples with whom it had no quarrel—the only offence of France was that she was the ally of Russia—which necessitated that Treaty. It is not for the good of the world that the health of Germany should suffer ; but it is not candid to seek to fasten the blame upon those who merely defended themselves from aggression, and a Germany which starved Paris into surrender is not entitled to complain of a " hunger-blockade."

### PUBLIC HEALTH IN CZECHOSLOVAKIA.

HOWEVER little we may admire the methods of the Czechoslovakian Goverment in dealing with other people's property, they must be given the fullest credit for their zeal for the public health—they are unwilling to allow even a great landowner to die of preventable disease. The republic begins at the beginning and publishes health pamphlets for children, and even fairy plays containing teaching relating to health, pleasantly disguised, no doubt, are provided. Lectures, films, and exhibitions dealing more or less immediately with hygiene are constantly being organised, and many popular illustrated pamphlets on sanitary subjects have been issued. School hygiene, tuberculosis, and the purity of milk have received the attention they deserve, and the Red Cross has been encouraged to supplement by voluntary effort a work which necessarily puts considerable strain upon the resources of the infant republic. There has even been an exhibition of the methods used in health education, and the Masaryk League Against Tuberculosis is conducting a very active propaganda. The Ministry of Health issues a " Popular Health Reader," and preparations are being made for a central State organisation of the various scattered activities for propaganda and training. It is to be hoped that some Governments a little farther East will not disdain to copy the methods of Bohemia.

### WASTED APPEALS.

ATTENTION is once more being called to the waste of effort involved in many charities with similar objects appealing simultaneously for help to a public which, broadly speaking, has no means of ascertaining the extent to which they deserve to be assisted. Owing to the exertions of the Ministry of Health Advisory Committee on the Welfare of the Blind, some five-and-twenty charities for the blind are now co-operating in their appeals, and the blind, as a whole, have benefited by this co-ordination. But why not go a step farther and amalgamate these efforts ? It is both absurd and irritating to the charitably disposed that effort should thus be split up. There are, we believe, seventy odd Societies in the Church of England all appealing for the poor clergy, and it is obvious that a great deal of money would be saved were there one central fund for this admirable purpose. Amalgamation, co-ordination, the doing of things *en large* are the notes of the time in matters of business ; why not in matters of charity also ?

### MEDICAL INSPECTION OF TEACHERS.

THE Education Committee at Carmarthen has passed a resolution recommending that all cases of tuberculosis, among teachers as well as scholars, in the schools of the county should be reported, examined and treated by a tuberculosis physician, and that the places of such teachers should be filled temporarily pending a medical report. The mover, Miss A. M. Davies, declared that a number of teachers were in this condition, and that the King Edward VII. Memorial Association should devote attention to preventive measures, and that the teachers, who were liable to infect the children, should receive appropriate treatment free of charge. The schools' medical officer should inspect doubtful cases, and report to the committee. It will be remembered that the County Council levies a rate for the benefit of the association, and therefore has an interest in seeing that the proceeds are wisely spent to arrest the disease at its source so far as possible. School medical inspection will necessarily remain incomplete, so long as the teachers remain outside the scope of the inspections.

### COUNTRY HOUSES AS INSTITUTIONS.

WAR taxation and the high cost of living are still bringing great numbers of fine old country houses into the market, and many of them have been, and are continuing to be, purchased for use as schools, hotels, and charitable institutions. Rendlesham Hall, Lord Rendlesham's great house in Suffolk, has lately been bought for institutional purposes, and it is now announced that Lord Tredegar has presented Cefyn Mably to the Welsh National Memorial Association for use as a hospital for tubercular patients. This beautiful old house, which is in Glamorganshire, not far from Cardiff, was for many centuries one of the seats of the family of Kemeys-Tynte, the head of which a few years ago made good his claim to the barony of Wharton, and of their ancestors in the female line. Although the place has been given a Georgian front and many of the rooms have been remodelled in the same taste, there are still architectural details which point to the time of Henry VII, or even earlier, and many of the bedrooms happily retain their Tudor features. In lovely and restful surroundings on the banks of the Rhymney the patients who will henceforth dwell beneath these venerable walls will enjoy what may fairly be called spiritual as well as material aids to recovery. Alike in sickness and in health, environment is an important influence for good, and we may well be thankful that Cefyn Mably is to be devoted to so admirable a purpose.

LECTURES AT THE ROYAL SANITARY INSTITUTE.—Two courses of training, the first for sanitary officers, the second for health visitors, school nurses, and maternity and child welfare workers, have been arranged by the Royal Sanitary Institute, and will begin on January 31 and February 2 respectively, at 6 p.m. These training courses are of particular interest just now, when so many educated women are being appointed on the staff of public health authorities, and the demand for trained women appears to be increasing. The training not only includes lectures, but practical demonstrations in the Museum of Sanitary Appliances, at Infant Consultations and Child Welfare Centres, visits to public works and other places of sanitary interest, and the use of a reference library, lending library and reading-room.

